# The Cost-effectiveness of Celecoxib versus Non-steroidal Anti-inflammatory Drugs plus Proton-pump Inhibitors for Treating Osteoarthritis in Algeria

**Published:** 2013-10-07

**Authors:** Nadir Hammoumraoui, Sid Ahmed Kherraf, Joaquin Mould-Quevedo, Tarek A. Ismail

**Affiliations:** 1 Rheumatology Department Etablissement Hospitalier Spécialisé Douera, Algiers, Algeria; 2 Algeria Business Center, Les Pins Maritimes, El Mohammadia, Algiers, Algeria; 3 Pfizer, Inc., New York, NY; 4 Pfizer, Inc., Dubai Media City, Dubai, UAE

**Keywords:** proton-pump inhibitors, algeria, cost-effectiveness, osteoarthritis, celecoxib

## Abstract

**Background:** Cyclooxygenase-2 inhibitors such as celecoxib are as effective as non-selective non-steroidal anti-inflammatory drugs (ns-NSAIDs) in the treatment of osteoarthritis (OA), have fewer gastrointestinal side effects, but are more expensive.

**Objective:** To evaluate the incremental cost-effectiveness ratio (ICER) of celecoxib versus ns-NSAIDs, with/without proton-pump inhibitor (PPI) co-therapy, for treating OA in Algeria.

**Methods:** The National Institute for Health and Clinical Excellence (NICE) health economic model from UK, updated with relative risks of adverse events using CONDOR trial data, was adapted for costeffectiveness analysis in OA patients aged ≥65 years. Patients could initiate treatment with celecoxib or ns-NSAIDs with/without omeprazole. Conditional probabilities were obtained from published clinical trials; effectiveness measure was quality-adjusted life years (QALYs) gained/patient. The analysis was conducted from a healthcare payer’s perspective. The average daily treatment costs and frequencies of resource use for adverse events were based on data collected in August 2011 from a private clinic located in Cheraga, Algiers, Algeria. Probabilistic sensitivity analysis (PSA) was performed to construct cost-effectiveness acceptability curves (CEACs).

**Results:** QALYs gained/patient over a 6-month horizon were higher with celecoxib (0.368) and celecoxib+PPI (0.40) versus comparators. The lowest expected cost/patient was associated with ibuprofen (US$134.76 versus US$175.67 with celecoxib+PPI, and US$177.57 with celecoxib). Celecoxib+PPI was the most cost-effective drug treatment, with an ICER of US$584.43, versus ibuprofen. Treatment with celecoxib alone showed an ICER of US$1,530.56 versus diclofenac+PPI. These ICERs are <1 gross domestic product per capita in Algeria (US$7,500). Over 1-year, 3-year and 5-year horizons, celecoxib with/without PPI co-therapy showed higher QALYs/patient versus comparators, and decreasing ICERs. The ICER of celecoxib+PPI was lower than that of comparators over all time horizons. These findings were confirmed with CEACs generated via PSA.

**Conclusion:** Using data from a single private clinic in Cheraga, Algiers, Algeria, and after considering new adverse event risks, we showed that celecoxib with/without PPI co therapy is more cost-effective than ns-NSAID+PPI for treating OA patients aged ≥65 years. Celecoxib+PPI remains dominant over a 5-year horizon, making it the most cost-effective treatment option for medium- and long-term use.

## 1. Introduction

Osteoarthritis (OA) is a degenerative condition of the cartilage characterized by joint pain leading to functional limitations and, consequently, disability and decreased quality of life.[1] The disease predominantly affects weight-bearing and stressed joints such as the knee, hip, and small joints of the hands.[1,2] The main risk factors for OA include age >40 years, female gender, positive family history, occupation and obesity.[3] Although the exact prevalence of OA is unknown, it is expected that incidence and prevalence will increase as life expectancy improves worldwide. Furthermore, the disease burden is likely to escalate in developing countries where life expectancy is high and constantly increasing, but there is inadequate access to surgical treatments.[4]

Pain relief with pharmacological therapy is an important component of managing OA. While paracetamol and topical non-steroidal anti-inflammatory drugs (NSAIDs) are the first line of treatment for OA, it is recommended to use oral NSAIDs including cyclooxygenase 2 (COX-2) inhibitors or opioid drugs, if first-line therapy fails to provide pain relief.[1]

As NSAID use is associated with gastrointestinal (GI) adverse events, it is recommended to combine these drugs with proton-pump inhibitors (PPIs), and choose an NSAID with the lowest acquisition cost.[1] A study performed by the National Institute for Health and Clinical Excellence (NICE) Osteoarthritis Guideline Development Group showed that addition of a PPI to an NSAID for patients with OA is cost-effective, even in those at low risk of GI adverse events. Of note, treatment with celecoxib plus PPI was more cost-effective than treatment with non-selective (ns)-NSAIDs plus PPI in patients at low and high risk of GI and cardiovascular (CV) adverse events.[5]

Studies have shown that selective NSAIDs such as COX-2 inhibitors are associated with less GI risk than ns-NSAIDs. In one study, the hazard ratio (HR) for hospitalization for ns-NSAIDS versus celecoxib was 2.28 (95% confidence interval [CI]: 1.82 to 2.61). Furthermore, the rate of hospitalization due to GI events was similar among patients prescribed ns-NSAIDs plus PPI and those prescribed celecoxib alone (HR: 0.96; 95% CI: 0.66 to 1.38).[6] In the Celecoxib versus Omeprazole and Diclofenac in patients with Osteoarthritis and Rheumatoid arthritis (CONDOR) study, significantly more GI events were observed in patients randomized to diclofenac plus omeprazole than in those prescribed celecoxib alone (HR: 4.3; 95% CI: 2.6 to 7.0; $p<0.0001$).[7]

Data show that OA is associated with substantial direct costs resulting from medications, treatment of comorbid conditions (related to osteoarthritis as well as ageing) and management of induced disability. It is also associated with indirect costs due to comorbidity and loss of productivity, which are difficult to measure.[8] Examination of the claims database of privately insured OA patients on pain medications in the United States found that the average annual direct medical, drug, and indirect work loss costs were US$8,601, US$2,941, and US$4,603, respectively.[9] Clearly, drug costs make a substantial contribution to the economic burden of OA. As celecoxib is more expensive than ns-NSAIDs, it is important to evaluate whether treatment with celecoxib is more cost-effective than that with ns-NSAIDs.

NICE is a special health authority that develops evidence-based guidance on public health, health technologies and clinical practice. A health economic model developed by NICE demonstrated that treatment with celecoxib plus PPI was more cost-effective than that with diclofenac plus PPI.[10] This economic model was based on data from three landmark randomized controlled trials, namely, the Celecoxib Long-term Arthritis Safety Study (CLASS; celecoxib, ibuprofen, and diclofenac),[11] the Multinational Etoricoxib and Diclofenac

Arthritis Long-term (MEDAL; etoricoxib and diclofenac) study[12] and the Therapeutic Arthritis Research and Gastrointestinal Event Trial (TARGET; lumiracoxib, naproxen and ibuprofen).[13,14] This NICE health economic model was recently updated with data from the CONDOR trial to show that when new adverse event risks were used, celecoxib plus PPI remained more cost-effective than diclofenac plus PPI.[15]

Although the exact prevalence of OA in Algeria is not known, the proportion of the population aged >60 years is expected to increase from 7.2% in 2012 to 26.2% in 2050.[16] This suggests that the population at risk of OA is likely to increase substantially in future, and this population will need pain medications to control OA symptoms. With this background, we conducted a study to evaluate the cost-effectiveness of celecoxib versus ns-NSAIDs, with or without PPI, in the treatment of OA in Algeria.

## 2. Methods

This cost-effectiveness analysis was conducted according to the methods recommended by NICE.[10] The primary outcome measure for effectiveness was quality adjusted life years (QALYs) gained/patient. Medical resource utilization and direct medical costs associated with each adverse event were calculated from the perspective of the third party payer which, for this study, was patients aged ≥65 years with OA in Algeria. This perspective was adopted as patients bear the cost of treatment for health conditions in Algeria.

### *Comparators*

Adverse event data were obtained from four large randomized control trials evaluating GI and CV events associated with NSAID use in patients with OA, namely, CLASS,[11] MEDAL,[12] TARGET,[13,14] and CONDOR.[7] Three of these studies (CLASS, MEDAL and CONDOR) also included a minority of patients with rheumatoid arthritis (RA). However, it was assumed that this factor would not bias the findings, as there is no evidence to link the rates of drug-induced adverse events with the type of arthritis.[5] This study compared the cost-effectiveness of celecoxib (200 mg BID) with the ns-NSAIDs naproxen (500 mg BID), diclofenac (75 mg BID) and ibuprofen (800 mg TID). In addition, the cost-effectiveness of adding the PPI omeprazole (20 mg) to each NSAID was also examined. These NSAIDs were chosen for analysis as there are sufficient data from clinical trials to draw reliable conclusions on the adverse events associated with these drugs. Furthermore, diclofenac, ibuprofen, naproxen and celecoxib are commonly prescribed for OA in Algeria.[17] Etoricoxib was eliminated from the analysis because it is not marketed in Algeria.

### *Model Design*

The NICE health economic model developed in 2008, and updated in 2012 with the relative risks of adverse events from the CONDOR trial was adapted for this study.[7,15] The economic model has been described elsewhere.[10] It is essentially a Markov model that compares the cost-effectiveness of individual ns-NSAIDs and COX-2 inhibitors based on the available adverse event data. The model assumes that ns-NSAIDs and COX-2 inhibitors are equally efficacious, and that different doses of the same drug are equally efficacious.[10] The model estimates the net impact of treatment on patient outcomes and expenditure, with due consideration to the incidence of GI and CV events, as well as improvement in OA symptoms. A theoretical cohort of patients is assumed to move between a series of health states and the rate of transition between states is estimated with the help of available clinical evidence.[5] This was done in accordance with the method followed by Latimer *et al*[5] and used by Brereton *et al*[15] when they adapted the NICE model using data from CONDOR. Within the model, patients do not switch treatments, except for the addition of PPI in the event of 'symptomatic ulcer' or 'complicated GI bleed'.[10]

The base case version of this model adopted a treatment duration of 6 months. The model assumed that patients initiated treatment with either celecoxib or ns-NSAID alone or in combination with a PPI. The length of the Markov cycle was set to last for 3 months.

The severe adverse events considered in the model included GI symptoms, symptomatic ulcer, complicated GI event, stroke, myocardial infarction and heart failure. In addition, patients may experience no adverse events or death. A patient may have only one GI or CV adverse event in each 3-month cycle.[10] A simplified version of the model structure is shown in Figure 1.

**Figure 1. Simplified Version of the Cost-effectiveness Model Structure**

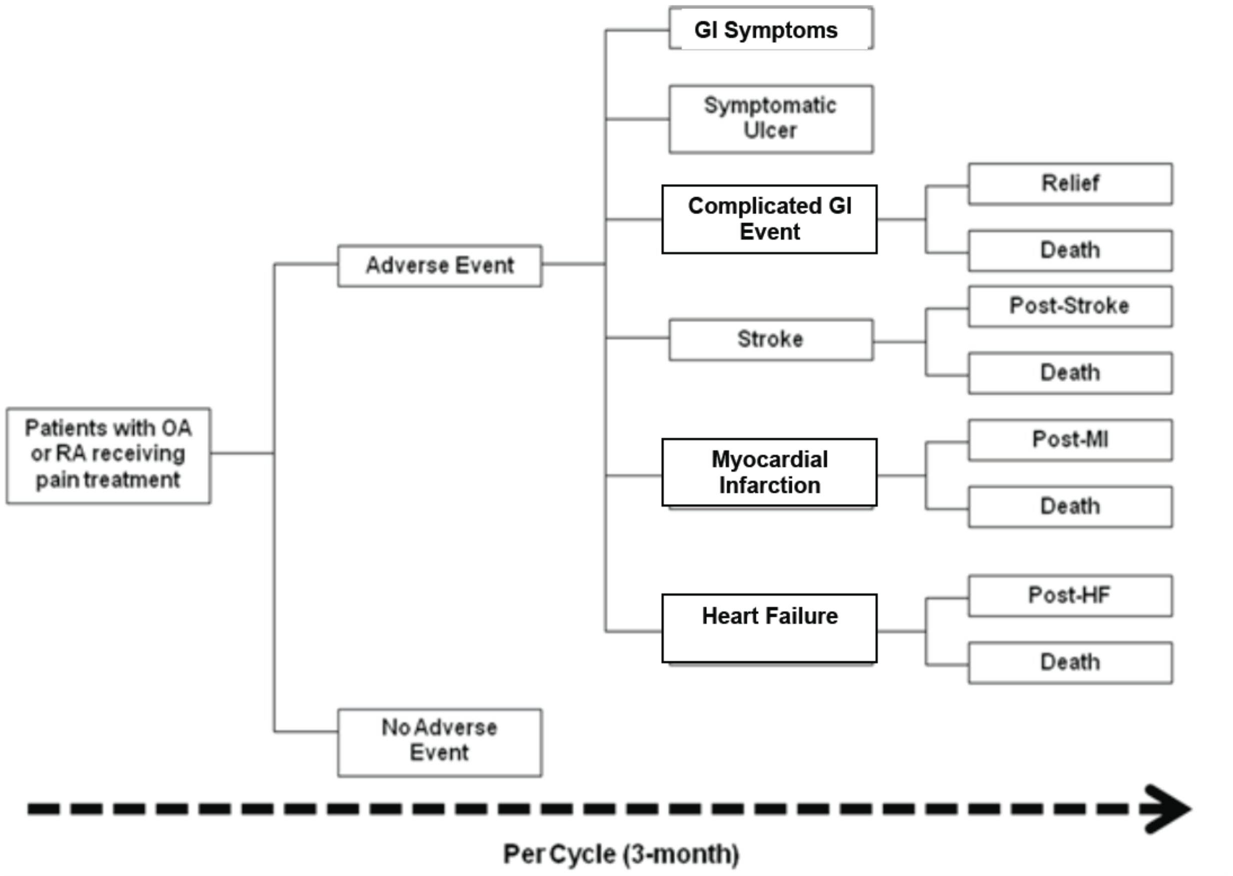

GI=gastrointestinal; OA=osteoarthritis; RA=rheumatoid arthritis; HF=heart failure

*Patient Populations*

The model estimated results for a cohort of patients with OA aged ≥65 years. The relative risk for each adverse event, calculated by Brereton *et al* by pooling data from the CLASS and CONDOR studies, was adapted for this study (Figure 2).[15]

**Figure 2. Relative Risks of Adverse Events based on Estimated Treatment Effects[15]**

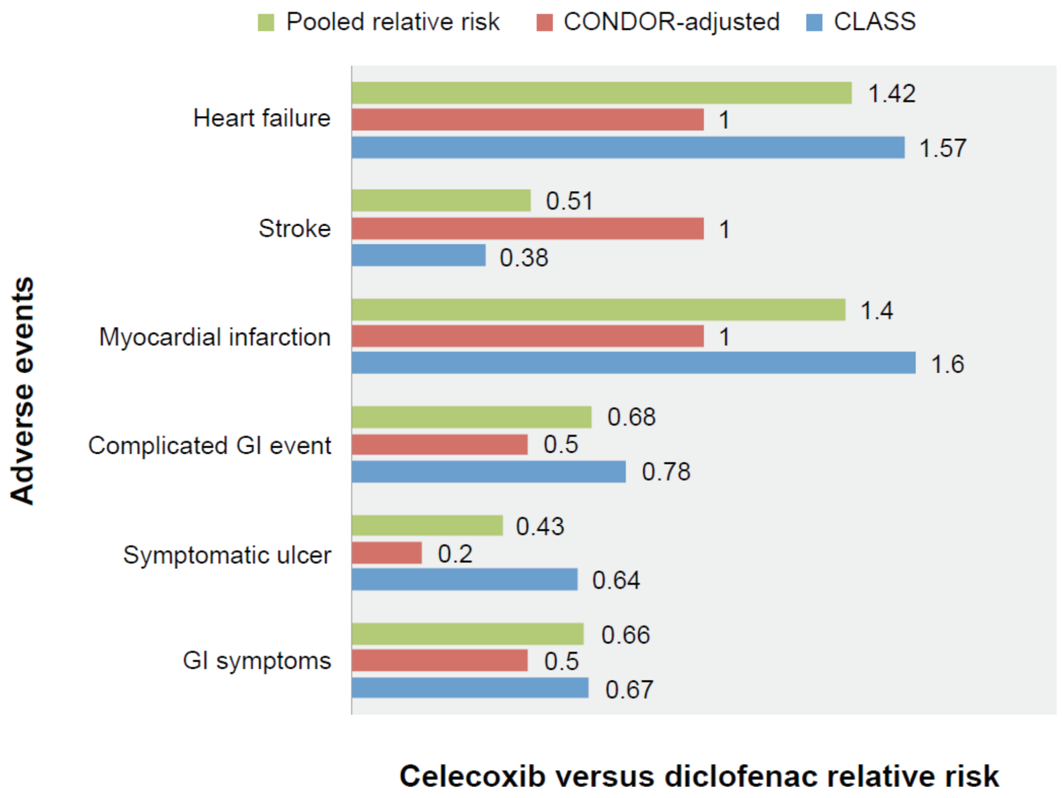

Adapted from Brereton *et al. J Med Econ* 2012;15(3):465-72; GI=gastrointestinal

*Adverse Events*

Adverse event data were obtained from the CONDOR, CLASS, MEDAL and TARGET studies.[7,11-14] As the doses used in these trials are similar to those used in clinical practice in Algeria, dose adjustment was not required. Figures 3 and 4 show the overall rates of adverse events observed with different NSAIDs in the four studies. Table 1 shows the transition probabilities per cycle associated with different treatment options. Transition probability refers to the risk of progressing to the next worst health state in the model within one cycle length. For this analysis, the progression is considered from a state of no symptoms through development of GI symptoms, symptomatic ulcer, complicated GI event, myocardial infarction, stroke and heart failure within a 3-month period.

*Costs*

Only direct medical costs incurred by patients were considered. These included costs of drugs, and costs of monitoring and treating adverse events associated with each drug. The latter comprised costs associated with hospitalization (including surgery for managing adverse events), outpatient procedures and consultations, and co-prescription of PPIs. The average daily treatment costs and frequencies of resource use for each adverse event were based on data collected in August 2011 from a private clinic located in Cheraga, Algiers, Algeria. The daily medication costs were obtained from IMS data collected in March 2013 (Table 2).[22]

**Figure 3. Overall Rates of Gastrointestinal Symptoms observed in CONDOR, MEDAL, CLASS and TARGET[7,11-14]**

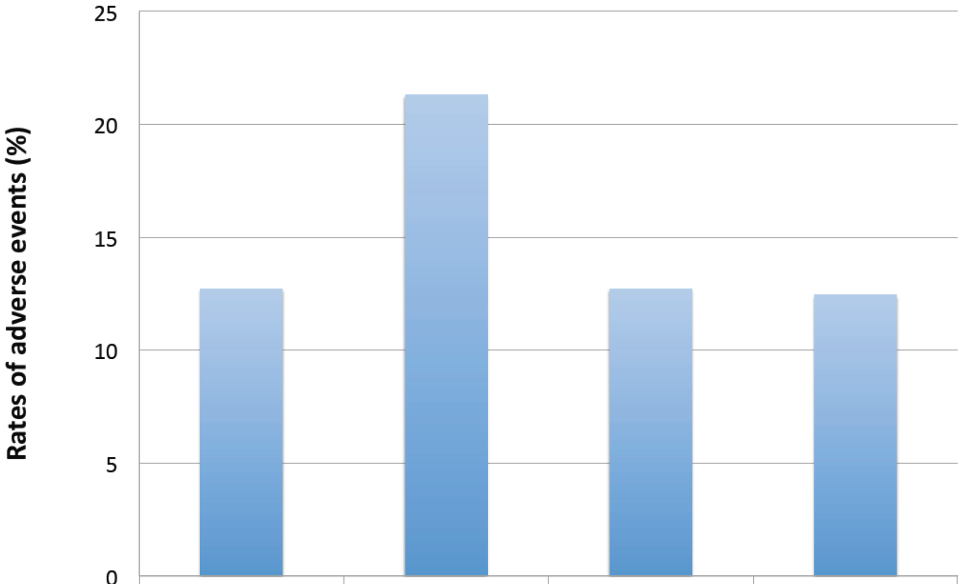

| | Paracetamol | Diclofenac | Ibuprofen | Celecoxib |
|---|---|---|---|---|
| GI symptoms | 12.72 | 21.3 | 12.72 | 12.45 |

GI=gastrointestinal

**Figure 4. Overall Rates of Adverse Events (excluding GI symptoms) observed in CONDOR, MEDAL, CLASS and TARGET[7,11-14]**

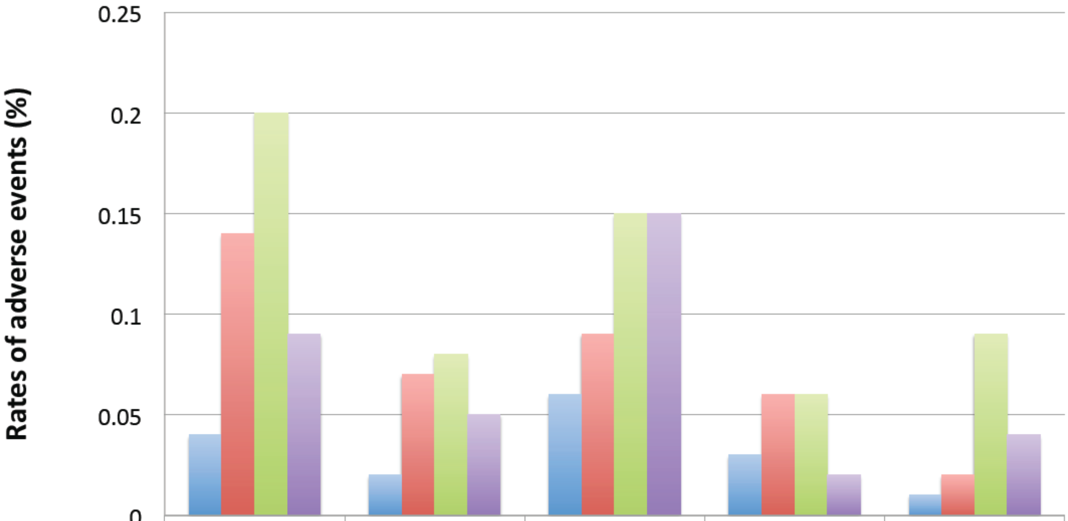

| | Symptomatic ulcer | Complicated GI event | Myocardial infarction | Stroke | Heart failure |
|---|---|---|---|---|---|
| Paracetamol | 0.04 | 0.02 | 0.06 | 0.03 | 0.01 |
| Diclofenac | 0.14 | 0.07 | 0.09 | 0.06 | 0.02 |
| Ibuprofen | 0.2 | 0.08 | 0.15 | 0.06 | 0.09 |
| Celecoxib | 0.09 | 0.05 | 0.15 | 0.02 | 0.04 |

GI=gastrointestinal

**Table 1. Transition Probabilities per Cycle with different Treatment Options for OA**

| Adverse Events | Transition Probabilities per Cycle | | | | | | | |
|---|---|---|---|---|---|---|---|---|
| | Paracetamol | Paracetamol + PPI | Diclofenac | Diclofenac + PPI | Ibuprofen | Ibuprofen + PPI | Celecoxib | Celecoxib + PPI |
| GI symptoms | 0.0853 | 0.0754 | 0.1339 | 0.0969 | 0.1616 | 0.1198 | 0.0671 | 0.0504 |
| Symptomatic Ulcer | 0.0006 | 0.0006 | 0.0012 | 0.0008 | 0.0014 | 0.0008 | 0.0006 | 0.0004 |
| Complicated GI event | 0.0003 | 0.0002 | 0.0006 | 0.0004 | 0.0006 | 0.0003 | 0.0004 | 0.0003 |
| Myocardial infarction | 0.0007 | 0.0007 | 0.0009 | 0.0009 | 0.0007 | 0.0007 | 0.0006 | 0.0006 |
| Stroke | 0.0003 | 0.0003 | 0.0003 | 0.0003 | 0.0003 | 0.0003 | 0.0003 | 0.0003 |
| Heart Failure | 0.0001 | 0.0001 | 0.0001 | 0.0001 | 0.0002 | 0.0002 | 0.0001 | 0.0001 |

Risk adjustment from CONDOR, CLASS, MEDAL, CLASS, TARGET7;[11-14] Data for adverse events were pooled from other published studies;[1,18-21] OA=osteoarthritis; PPI=proton pump inhibitor; GI=gastrintestinal

On average, it was assumed that patients made one consultation per adverse event. Patients with dyspepsia or symptomatic ulcer were considered to need only outpatient management. Hospitalization costs were considered in patients with complicated GI event, myocardial infarction stroke and heart failure, with the duration of hospitalization determined by the type and severity of the adverse event.

Costs were estimated for treatment over a period of 6 months, 1 year, 3 years and 5 years. The costs of managing adverse events and resource utilization were assumed to be the same for each drug. All daily cost data were expressed in 2013 prices and converted to US$ using an average 2013 exchange rate (1 Algerian Dinar = US$0.013). All costs and events were discounted at 3% annually to account for the current value.

**Table 2. Daily Costs of NSAIDs in Algeria[22]**

| Drug and Dose | Cost (US$)* |
|---|---|
| Diclofenac 50 mg TID | 0.09 |
| Celecoxib 100 mg QD | 0.34 |
| Celecoxib 200 mg BID | 0.68 |
| Celecoxib 300 mg QD | 0.68 |
| Naproxen 275 mg BID | 0.12 |
| Naproxen 550 mg OD | 0.24 |
| Ibuprofen 400 mg TID | 0.11 |
| Omeprazole 20 mg OD | 0.18 |

*1 Algerian Dinar = 0.013 US$ in 2013; NSAIDs=non-steroidal anti-inflammatory drugs

*QALY Data*

The EurQol (EQ)-5D and Western Ontario and McMaster Universities Osteoarthritis Index (WOMAC) scores were used to predict utility scores. EQ-5D is a standardized instrument used to measure health outcomes, while WOMAC is a 24-item instrument validated for assessing pain, stiffness and physical function in patients with OA, and is widely used for evaluating outcomes in OA clinical trials.[23,24]

A meta-analysis of total WOMAC scores for each drug was conducted, and transformed into EQ-5D scores using the 'transfer of utility' mapping technique.[25] Finally, the EQ-5D scores were used to estimate quality of life weights reflecting improvements in symptom control. The meta-analysis found no significant differences between the efficacy of COX-2 inhibitors and ns-NSAIDs. In addition, there was no evidence of different efficacy related to different doses of medications. Thus, for this study, equal utility weights were assumed for ns-NSAIDS and COX-2 inhibitors in patients who did not experience adverse events. However, paracetamol was assigned a lower utility weight as its efficacy was inferior to both ns-NSAIDS and COX-2 inhibitors. Utility weights for adverse events were assigned based on evidence from the literature.[5]

*Cost-effectiveness Analyses*

The expected costs and the estimated QALY gain associated with eight treatment options were calculated over a period of 6 months, 1 year, 3 years and 5 years. Incremental cost effectiveness ratios (ICERs), defined as the additional cost per patient achieving a unit of effectiveness compared with the next less costly, non-dominated option, were calculated for each time horizon.

*Sensitivity Analyses*

The robustness of the model was tested with probabilistic sensitivity analysis using Monte Carlo simulations. This involved generating 10,000 second-order iterations of the analysis. For each iteration, a different set of values was used for the parameters selected for the analysis. The 10,000 iterations generated costs and QALY results which were used to construct a cost-effectiveness acceptability curve. This curve served to demonstrate the confidence with ICERs obtained over a range of thresholds of acceptability.

## 3. Results

*Base Case Analysis*

Base case analysis, ie, treatment over a period of 6 months, showed that the estimated effectiveness in terms of QALYs gained per patient was higher with celecoxib (0.368 QALYs/patient) and celecoxib plus PPI (0.40 QALYs/patient) versus all comparators for treating OA patients aged ≥65 years. The expected cost of treatment with these treatments was also higher versus comparators, with the lowest expected cost associated with ibuprofen (cost per patient, US$134.76 versus US$175.67 with celecoxib plus PPI, and US$177.57 with celecoxib) (Table 3).

The most cost-effective option for OA treatment over a 6-month period was celecoxib plus PPI with an incremental cost of US$40.91 per patient, and an incremental QALY gain of 0.07 per patient, resulting in an ICER of US$584.40, versus ibuprofen. Treatment with naproxen (with or without PPI), diclofenac (with or without PPI), and celecoxib were simultaneously more expensive and less effective than comparators, and thus subject to simple dominance. Ibuprofen plus PPI and diclofenac plus PPI were eliminated by extended dominance, as these had higher ICERs than celecoxib plus PPI (Table 3).

When celecoxib plus PPI was excluded from the analysis, the incremental cost and QALY gained per patient with celecoxib alone were US$27.55 and 0.02, respectively, giving an ICER of US$1,530.56 versus diclofenac plus PPI (data not shown). This ICER is less than US$7,500, which is the estimated GDP per capita in Algeria.[26] According to the Commission on Macroeconomics and Health of the World Health Organization (WHO), the threshold for highly cost-effective interventions is an ICER less than one GDP

per capita of a country.[27] Thus, both celecoxib plus PPI and celecoxib alone are highly effective cost-effective options for treating OA in Algeria.

**Table 3. Cost Effectiveness over a 6-month Time Horizon: Base Case Analysis**

| Treatment | Cost/Patient (US$) | QALYs Gained/Patient | ICER (US$) | Comparator |
|---|---|---|---|---|
| Ibuprofen | 134.76 | 0.330 | 408.36 | No treatment |
| Ibuprofen + PPI* | 147.66 | 0.343 | Dominated | |
| Diclofenac + PPI* | 150.02 | 0.350 | Dominated | |
| Naproxen + PPI§ | 156.69 | 0.338 | Dominated | |
| Diclofenac§ | 160.11 | 0.305 | Dominated | |
| Naproxen§ | 167.68 | 0.280 | Dominated | |
| Celecoxib + PPI | 175.67 | 0.400 | 584.43 | Ibuprofen |
| Celecoxib§ | 177.57 | 0.368 | Dominated | |

QALYs=quality-adjusted life years; ICER=incremental cost-effectiveness ratio; PPI=proton pump inhibitor

§Treatment subject to simple dominance, ie, another option is less expensive and more effective

*Treatment subject to extended dominance, ie, a combination of two other options is less expensive and more effective

ICER: Additional cost per additional QALY gained, comparing each non-dominated option with the next most expensive, non-dominated option

*Sensitivity Analyses*

Analyses on the basis of treatment over 1-year, 3-year and 5-year time horizons showed that celecoxib alone and celecoxib plus PPI treatments were associated with persistently higher QALYs per patient versus comparators, and decreasing ICERs. Of note, the ICER of celecoxib plus PPI remained persistently lower than that of comparators over all time horizons (Tables 4, 5 and 6). Over a 5-year time horizon, celecoxib plus PPI was associated with an expected incremental cost per patient of US$22.70, and an incremental QALY gain of 0.29 per patient, resulting in an ICER of US$79.65 versus ibuprofen plus PPI (Table 6).

**Table 4. Cost Effectiveness Analysis over a 1-year Time Horizon**

| Treatment | Cost/Patient (US$) | QALYs Gained/Patient | ICER (US$) | Comparator |
|---|---|---|---|---|
| Ibuprofen | 266.67 | 0.60 | 448.18 | No treatment |
| Ibuprofen + PPI | 270.77 | 0.65 | 70.69 | Ibuprofen |
| Diclofenac + PPI* | 277.76 | 0.67 | Dominated | |
| Naproxen + PPI§ | 292.05 | 0.64 | Dominated | |
| Diclofenac§ | 303.52 | 0.56 | Dominated | |
| Celecoxib + PPI | 316.87 | 0.78 | 377.87 | Ibuprofen + PPI |
| Naproxen§ | 320.96 | 0.50 | Dominated | |
| Celecoxib§ | 325.80 | 0.71 | Dominated | |

QALYs=quality-adjusted life years; ICER=incremental cost-effectiveness ratio; PPI=proton pump inhibitor

§Treatment subject to simple dominance, ie, another option is less expensive and more effective

*Treatment subject to extended dominance, ie, a combination of two other options is less expensive and more effective

ICER: Additional cost per additional QALY gained comparing each non-dominated option with the next most expensive, non-dominated option.

**Table 5. Cost Effectiveness Analysis over a 3-year Time Horizon**

| Treatment | Cost/Patient (US$) | QALYs Gained/Patient | ICER (US$) | Comparator |
|---|---|---|---|---|
| Ibuprofen + PPI | 590.67 | 1.34 | 440.80 | No treatment |
| Diclofenac + PPI* | 608.50 | 1.36 | Dominated | |
| Celecoxib + PPI | 641.74 | 1.57 | 219.18 | Ibuprofen + PPI |
| Ibuprofen§ | 647.93 | 1.17 | Dominated | |
| Naproxen + PPI§ | 654.47 | 1.30 | Dominated | |
| Celecoxib§ | 693.99 | 1.44 | Dominated | |
| Diclofenac§ | 721.02 | 1.13 | Dominated | |
| Naproxen§ | 796.01 | 1.00 | Dominated | |

QALYs=quality-adjusted life years; ICER=incremental cost-effectiveness ratio; PPI=proton pump inhibitor

§Treatment subject to simple dominance, ie, another option is less expensive and more effective

*Treatment subject to extended dominance, ie, a combination of two other options is less expensive and more effective

ICER: Additional cost per additional QALY gained comparing each non-dominated option with the next most expensive, non-dominated option

**Table 6. Cost Effectiveness Analysis over a 5-year Time Horizon**

| Treatment | Cost/Patient (US$) | QALYs Gained/Patient | ICER (US$) | Comparator |
|---|---|---|---|---|
| Ibuprofen + PPI | 745.98 | 2.39 | 312.13 | No treatment |
| Diclofenac + PPI§ | 767.99 | 2.39 | Dominated | |
| Celecoxib + PPI | 768.68 | 2.68 | 79.65 | Ibuprofen + PPI |
| Naproxen + PPI§ | 839.84 | 2.31 | Dominated | |
| Celecoxib§ | 859.00 | 2.50 | Dominated | |
| Ibuprofen§ | 865.50 | 2.08 | Dominated | |
| Diclofenac§ | 967.81 | 2.05 | Dominated | |
| Naproxen§ | 1,108.33 | 1.83 | Dominated | |

QALY=quality-adjusted life years; ICER=incremental cost-effectiveness ratio; PPI=proton pump inhibitor

§Treatment subject to simple dominance, ie, another option is less expensive and more effective

ICER: additional cost per additional QALY gained comparing each non-dominated option with the next most expensive, non-dominated option

After excluding celecoxib plus PPI from the analysis, the expected incremental cost and QALY gained per patient over a 5-year time horizon with celecoxib alone were US$113.02 and 0.11, respectively, resulting in an ICER of US$1,027.45 versus ibuprofen plus PPI (data not shown). As this value is less than one GDP per capita in Algeria, celecoxib alone is considered a highly effective intervention for long-term treatment of OA in Algeria.

The robustness of these findings was confirmed with the cost-effectiveness acceptability curves generated via probabilistic sensitivity analysis shown in Figures 5 and 6. For a given cost effectiveness threshold selected on the horizontal axis, the cumulative proportion of the 10,000 simulations that resulted in a cost-effectiveness ratio equal to or less than the specified threshold can be read from the vertical axis.

Over a 5-year treatment horizon, there was 100% probability that celecoxib plus PPI as well as celecoxib alone would be the most cost-effective treatment options versus ns-NSAIDs with or without PPI co-therapy for OA patients aged ≥65 years, at a cost-effectiveness threshold of less than one GDP per capita in Algeria.[27]

**Figure 5. Cost Effectiveness Acceptability Curves on the basis of 5 years of Treatment: Celecoxib plus PPI versus Celecoxib Alone and ns-NSAIDs with and without PPI Co-therapy**

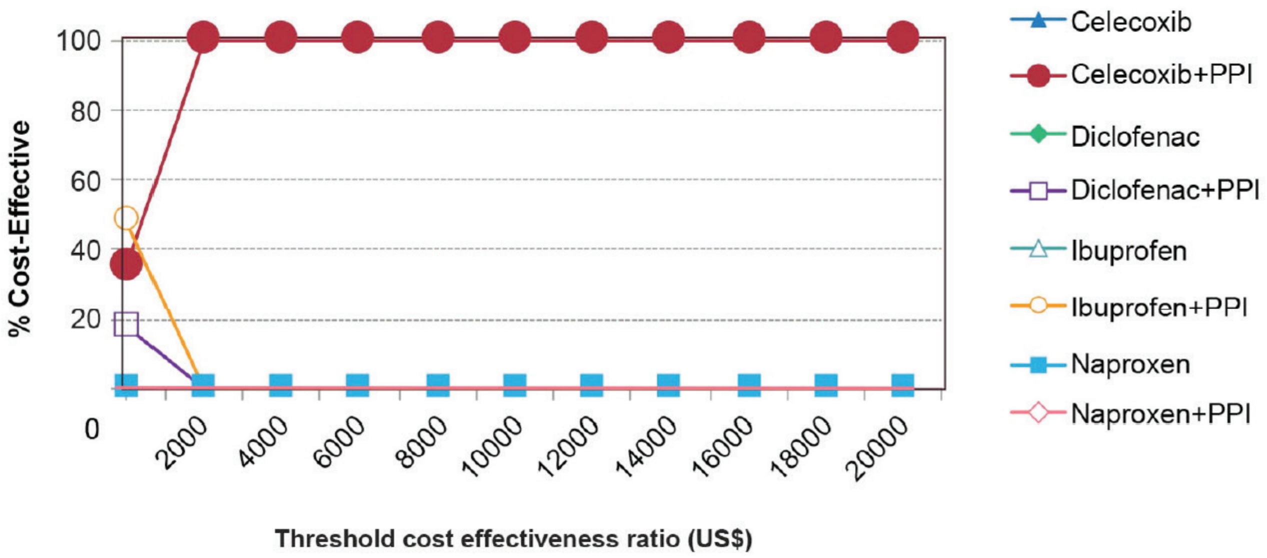

ns-NSAIDs=non-selective non-steroidal anti-inflammatory drugs; PPI=proton-pump inhibitor

**Figure 6. Cost Effectiveness Acceptability Curves on the basis of 5 years of Treatment: Celecoxib alone versus ns-NSAIDs with and without PPI Co-therapy**

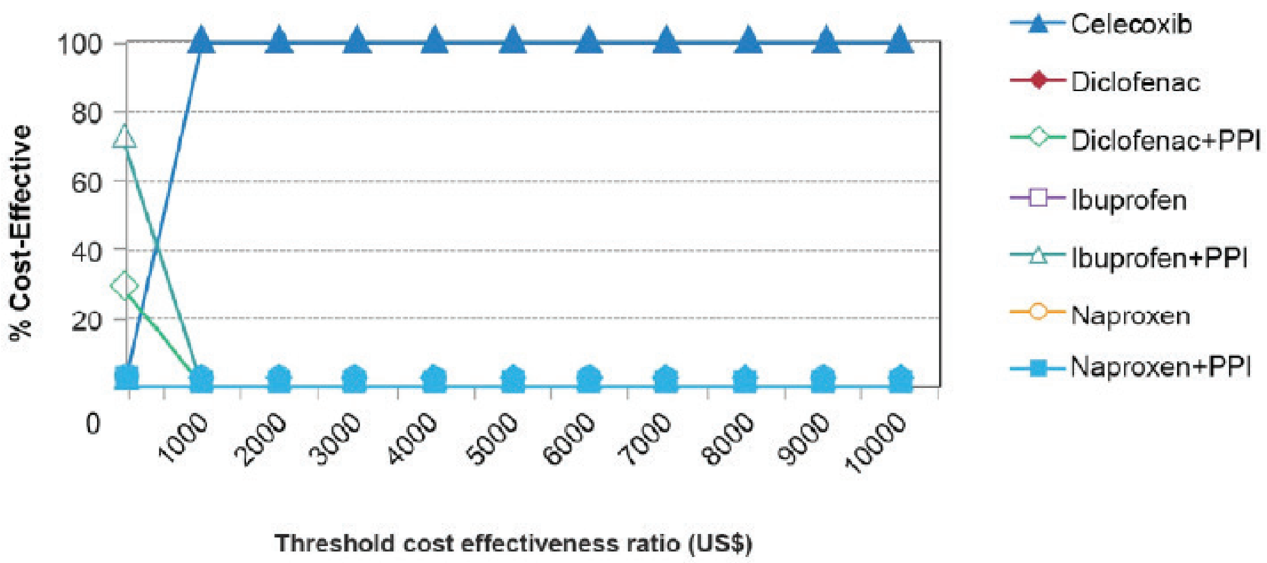

ns-NSAIDs=non-selective non-steroidal anti-inflammatory drugs; PPI=proton-pump inhibitor

## 4. Discussion

Using the adverse event data from four large randomized trials among patients predominantly with OA, this cost-effectiveness analysis found that, over a time frame of 6 months, celecoxib plus PPI is more cost effective than ns-NSAIDs, with or without PPI co-therapy, for OA patients aged ≥65 years in Algeria. Given the wide prevalence of OA, the need for pain relief in the selected patient population and the fact that patients bear the cost of treatment, it is important to choose an analgesic which has a proven efficacy and safety profile and is also cost effective.

Based on the threshold of one GDP per capita in Algeria prescribed by the WHO, celecoxib plus PPI as well as celecoxib alone were highly cost-effective options for treating OA in Algeria. Furthermore, over a period of 5 years, treatment with celecoxib plus PPI was expected to remain the most cost-effective option in this patient population versus ns-NSAIDs with or without PPI co-therapy. Second to celecoxib plus PPI, treatment with celecoxib alone was expected to have the highest QALY gained per patient over 5 years, and an ICER well within the WHO-prescribed cost-effectiveness ratio threshold in Algeria.

These results resonate with those from a recent evaluation by Brereton *et al*, who used the same economic model and clinical data to show that celecoxib plus PPI was more cost-effective than diclofenac plus PPI for treating OA in the United Kingdom. After pooling data from the CLASS and CONDOR trials, they showed that celecoxib plus PPI was associated with an ICER of £9,377 per QALY versus diclofenac plus PPI.[15]

Findings from this analysis also agree with those from numerous other studies evaluating the cost-effectiveness of celecoxib alone or in combination with PPI for treating arthritis.[5,28-31] Latimer *et al* found that addition of PPI to a COX-2 inhibitor was associated with an ICER of £10,000, which was cost-effective even for OA patients at low risk of GI events.[5] Two cost-effectiveness analyses performed from a public health organization perspective in Hong Kong concluded that celecoxib was more cost-effective than ns-NSAIDs with or without PPI co-therapy for treating arthritis patients with high GI risk.[29,30] Contreras *et al* conducted a cost-effectiveness analysis from a Mexican institutional perspective, which showed that celecoxib was associated with the lowest cost-effectiveness ratio of US$1.75 per patient, when compared with ns-NSAIDs and acetaminophen. Celecoxib was associated with a low rate of adverse events and, consequently, low use of resources and decreased healthcare costs.[31]

The strength of this analysis is that celecoxib plus PPI was shown to be the most cost-effective option despite consideration of possible associated CV side effects such as myocardial infarction, stroke and heart failure. It is important to consider CV risk in such analyses as the majority of OA patients are elderly and have cardiovascular conditions. A cost-effectiveness analysis by Schaefer *et al*, modelled according to the Veteran Health Administration perspective in the United States, found that treatment with both celecoxib and rofecoxib was more cost effective than ns-NSAIDs in high-risk populations (ie, patients aged ≥65 years and with a previous history of perforation, ulcer or bleed). However, the risk of CV complications was greater with rofecoxib versus celecoxib. Consequently, the cost per QALY gained was less than US$50,000 with celecoxib, while treatment with rofecoxib was more expensive and associated with net QALY loss.[32]

The other strength of this analysis is the period of 6 months for base case analysis. As OA is a chronic condition, patients use pain medications over long periods of time. Thus, it is important to know the cost-effectiveness of drugs over long-term use. Findings from the base case analysis were used for extrapolating the impact of treatments with eight different therapies over a long time horizon (ie, 5 years). Furthermore, cost-effectiveness acceptability curves over a 5-year time horizon confirmed the robustness and reliability of the study findings. Previous cost effectiveness studies assessed short, 3-month periods of treatment, which may not reflect real-world use of NSAIDs in OA patients.[5,15]

This analysis has some limitations. Data show that the risk of bleeding associated with NSAID use increases with rising age, and celecoxib has been shown to be a cost-effective option over long time frames for patients aged >60 years who have at least an average baseline risk of GI bleed.[33] In this analysis, the ICER for celecoxib plus PPI decreased over time, and was as low as US$ 79.65 versus ibuprofen plus PPI over a 5-year time horizon. This finding may be considered to align with the factor of increased age-associated GI risk. However, we did not perform a subgroup analysis to investigate whether patients aged ≥65 years with baseline risk of GI bleed had more benefits with celecoxib plus PPI versus those without risk of GI bleed.[5] In their cost-effectiveness analysis, Schaefer *et al* demonstrated that treatment with celecoxib was more cost-effective in patients with a history of GI bleed than in those aged ≥65 years without such history.[32] Similarly, El Serag et al noted that it was cost-effective to use celecoxib despite its relatively high cost, or to add a PPI to a ns-NSAID, in patients with moderately high risk of upper GI bleeding.[34] In this analysis, celecoxib alone was more effective than ns-NSAIDs with or without PPI co-therapy, in terms of QALYs gained per patient. However, celecoxib was dominated by celecoxib plus PPI across all time horizons. It would have been interesting to know if there was a population of patients who had high baseline risk of GI bleed and to evaluate the economic benefits of celecoxib alone in this population.

The other limitation is that only direct medical costs were considered in this analysis as it was not feasible to obtain data on the indirect costs associated with OA in Algeria. It would have been a more comprehensive cost-effectiveness analysis if both direct and indirect costs had been considered.

Finally, as the source of adverse event data was limited to four clinical trials, there is a possibility that the incidence of adverse events may not reflect that in the general population. However, all four studies used as data sources were large, randomized controlled trials conducted in multiple centres across several countries around the world.[7,11-14] Thus, it is reasonable to assume that these data reflect real-life frequencies of adverse events.

## 5. Conclusions

Using data from a single private clinic in Cheraga, Algiers, Algeria, this cost-effectiveness analysis demonstrated that celecoxib plus PPI and celecoxib alone are more cost effective than ns-NSAIDs plus PPI for treating OA patients aged ≥65 years in Algeria, even after considering new adverse event risks. Of note, celecoxib plus PPI remained a dominant option over a 5-year time horizon, making it the most cost-effective treatment option for medium- and long-term use in this patient population.

**Acknowledgements**

The authors thank Dr. Pradnya Kulkarni from MIMS (Hong Kong) Limited for providing editorial support.

**Conflict of Interest Declaration**

This study was funded by Pfizer, Inc. Co-authors Joaquin Mould-Quevedo, Sid Ahmed Kherraf and Ismail A. Tarek are full-time employees of Pfizer, Inc.
